# The effect of intravenous golimumab on health-related quality of life and work productivity in patients with active psoriatic arthritis: results of the Phase 3 GO-VIBRANT trial

**DOI:** 10.1007/s10067-021-05639-1

**Published:** 2021-03-02

**Authors:** Alexis Ogdie, Jessica A. Walsh, Soumya D. Chakravarty, Steven Peterson, Kim Hung Lo, Lilianne Kim, Nan Li, Elizabeth C. Hsia, Eric K. H. Chan, Arthur Kavanaugh, M. Elaine Husni

**Affiliations:** 1 University of Pennsylvania, 3400 Spruce St, White Building, Room 5023, Philadelphia, PA 19104 USA; 2 University of Utah, George E. Wahlen Veterans Affairs, Salt Lake City, UT USA; 3 Janssen Scientific Affairs, LLC, Horsham, PA USA; 4 Drexel University College of Medicine, Philadelphia, PA USA; 5 Janssen Global Services, LLC, Horsham, PA USA; 6 Janssen Research & Development, LLC, Spring House, PA USA; 7 Janssen Global Services, LLC, Raritan, NJ USA; 8 University of California–San Diego, San Diego, CA USA; 9 Cleveland Clinic, Cleveland, OH USA

**Keywords:** Health-related quality of life, Intravenous golimumab, Productivity, Psoriatic arthritis

## Abstract

**Introduction/objectives:**

To evaluate changes in health-related quality of life (HRQoL) and productivity following treatment with intravenous (IV) golimumab in patients with psoriatic arthritis (PsA).

**Methods:**

Patients were randomized to IV golimumab 2 mg/kg (*n*=241) at Weeks 0, 4, then every 8 weeks (q8w) through Week 52 or placebo (*n*=239) at Weeks 0, 4, then q8w, with crossover to IV golimumab 2 mg/kg at Weeks 24, 28, then q8w through Week 52. Change from baseline in EuroQol-5 dimension-5 level (EQ-5D-5L) index and visual analog scale (EQ-VAS), daily productivity VAS, and the Work Limitations Questionnaire (WLQ) was assessed. Relationships between these outcomes and disease activity and patient functional capability were evaluated post hoc.

**Results:**

At Week 8, change from baseline in EQ-5D-5L index (0.14 vs 0.04), EQ-VAS (17.16 vs 3.69), daily productivity VAS (−2.91 vs −0.71), and WLQ productivity loss score (−2.92 vs −0.78) was greater in the golimumab group versus the placebo group, respectively. At Week 52, change from baseline was similar in the golimumab and placebo-crossover groups (EQ-5D-5L index: 0.17 and 0.15; EQ-VAS: 21.61 and 20.84; daily productivity VAS: −2.89 and −3.31; WLQ productivity loss: −4.49 and −3.28, respectively). HRQoL and productivity were generally associated with disease activity and functional capability, with continued association from Week 8 through Week 52.

**Conclusion:**

IV golimumab resulted in early and sustained improvements in HRQoL and productivity from Week 8 through 1 year in patients with PsA. HRQoL and productivity improvements were associated with improvements in disease activity and patient functional capability.

**Table:** 

**Key Points** • *In patients with active psoriatic arthritis (PsA), intravenous (IV) golimumab improved health-related quality of life (HRQoL) and productivity as early as 8 weeks and maintained improvement through 1 year* • *Improvements in HRQoL and productivity outcomes in patients with PsA treated with IV golimumab were associated with improvements in disease activity and patient functional capability outcomes* • *IV golimumab is an effective treatment option for PsA that can mitigate the negative effects of the disease on HRQoL and productivity*

**Supplementary Information:**

The online version contains supplementary material available at 10.1007/s10067-021-05639-1.

## Introduction

Psoriatic arthritis (PsA) is a chronic inflammatory disease characterized by peripheral joint inflammation, enthesitis, dactylitis, spondylitis, psoriatic skin lesions, and nail psoriasis [1]. In addition to the multifaceted musculoskeletal and cutaneous burden, progressive joint deformity can result in significantly reduced health-related quality of life (HRQoL), functional impairment in performing daily and work-related activities, and disability [2–7]. Functional impairment can have substantial socioeconomic implications and is an important outcome measure in PsA [7, 8].

In a longitudinal analysis conducted over 10 years, 72% of patients with PsA experienced disability [9]. Health-related limitations at work, including absenteeism and reduced effectiveness, have been reported in 16 to 49% of patients with psoriasis or PsA [10–12]. In one report, work productivity, as measured by the Work Limitations Questionnaire (WLQ), was reduced by 7% in patients with PsA compared with benchmark employees without limitations [10]. In another multinational, real-world PsA population, the presence of enthesitis, dactylitis, inflammatory back pain, or sacroiliitis was significantly associated with worse patient quality of life and/or work productivity as measured by the EuroQol-5 dimension-5 level (EQ-5D-5L) index and visual analog scale (EQ-VAS) and the work productivity and activity index [13]. Thus, an important goal of PsA treatment is improved HRQoL and productivity.

In GO-VIBRANT, a Phase 3 randomized, placebo-controlled trial, intravenous (IV) golimumab, a fully human monoclonal antitumor necrosis factor (TNF) antibody, was both safe and efficacious, with sustained improvements in both joint and skin symptoms through 1 year in patients with active PsA [14, 15]. Additionally, improvements in physical function and HRQoL, evaluated using the Health Assessment Questionnaire-Disability Index (HAQ-DI), Short Form 36 Health Survey (SF-36) physical component summary (PCS) and mental component summary (MCS), EQ-VAS, Functional Assessment of Chronic Illness Therapy-Fatigue (FACIT-F), and Dermatology Life Quality Index (DLQI), were greater with golimumab versus placebo as early as Week 8 and through Week 24 [16]. Further, IV golimumab treatment has been shown to improve HRQoL, as well as work productivity in patients with the related spondyloarthritis, ankylosing spondylitis [17].

The aim of this post hoc analysis was to examine the long-term effects of IV golimumab on patient-reported outcome measures evaluating HRQoL (EQ-5D-5L index and EQ-VAS) through 1 year in patients with active PsA who participated in the GO-VIBRANT trial and to also evaluate changes in daily and work productivity in these patients. Additionally, we examined whether improvement in measures of HRQoL and productivity were associated with underlying improvement in measures of overall disease activity and patient functional capability.

## Materials and methods

### Patients

Details of the eligibility criteria and trial design of the GO-VIBRANT trial were described previously [14]. Briefly, biologic-naïve patients ≥18 years of age diagnosed with PsA for at least 6 months at screening based on ClASsification criteria for Psoriatic Arthritis (CASPAR) criteria [18] were included in the GO-VIBRANT trial. Patients had active PsA, defined as ≥5 swollen and ≥5 tender joints at screening and baseline and a C-reactive protein (CRP) level ≥0.6 mg/dL at screening, despite current or previous treatment with disease-modifying antirheumatic drugs (≥3 months) and/or nonsteroidal anti-inflammatory drugs (≥4 weeks). All patients provided written informed consent.

### Trial design

Eligible patients were randomized to receive IV golimumab 2 mg/kg at Weeks 0 and 4 and then every 8 weeks (q8w) through Week 52 or placebo (normal saline for IV infusion) at Weeks 0 and 4 and then q8w, with crossover to IV golimumab 2 mg/kg at Weeks 24 and 28 and then q8w through Week 52 [14]. At Week 16, patients in either treatment group who qualified for early escape (<5% improvement in swollen and tender joint counts) were allowed to receive a protocol-specified change in concomitant medications at the investigator’s discretion.

This trial was registered with clinicaltrials.gov (NCT02181673). The trial protocol was approved by an institutional review board or local ethics committee for each site, and the trial was conducted in accordance with the principles of the Declaration of Helsinki that are consistent with Good Clinical Practices and local regulatory requirements.

### Trial assessments

General health status was measured using the United States model of the EQ-5D-5L standardized measure of health status, which is a descriptive system that comprises 5 dimensions—mobility, self-care, usual activities, pain/discomfort, and anxiety/depression [19]. Each dimension has 5 levels: no problems, slight problems, moderate problems, severe problems, and extreme problems. Each respondent was asked to indicate their health state in each of the 5 individual dimensions; a decrease in dimension score indicates improvement. The dimension scores were converted into a single summary index (EQ-5D-5L index) by applying a formula that attaches values (also called weights) to all possible combinations of levels in each dimension [20, 21]. The resulting score represents overall utility or general HRQoL, with 1 representing perfect health, 0 representing death, and negative values representing a state worse than death. The EQ-5D-5L also has a VAS element (EQ-VAS), which records the respondent’s answer to the question “How good or bad is your health today?” on a vertical VAS where the endpoints are labeled “worst imaginable health state” (0) and “best imaginable health state” (100); an increase in score indicates improvement.

Health-related productivity loss was evaluated using a daily productivity VAS and the WLQ. The daily productivity VAS records the respondent’s answer to the question “How much has your disease affected your daily productivity at work, school, or home in the past 4 weeks” on a horizontal VAS where the endpoints are labeled “did not affect my productivity at all” (scored as 0) and “affected my productivity very much” (scored as 10); a decrease in score indicates improvement. The WLQ, which was administered only to patients who were working full or part time, including volunteering, is a 25-item self-report questionnaire that asks respondents to rate their level of difficulty or ability to perform specific job demands [22]. The 25 items are aggregated into 4 domains—time management, physical demands, mental–interpersonal, and output. Scale scores for each domain range from 0 (limited none of the time) to 100 (limited all of the time); a decrease in domain score indicates improvement. The 4 domains were also converted into an estimate of productivity loss (WLQ productivity loss score); a decrease in score indicates improvement.

Disease activity was assessed using the Psoriasis Area and Severity Index (PASI) [23], a modified version of the Disease Activity Score including 28 joints (DAS28) [24], and Disease Activity index for PSoriatic Arthritis (DAPSA) [25]. DAS28 is a statistically derived index combining 4 disease assessments (tender joint count [TJC; 28 joints], swollen joint count [SJC; 28 joints], CRP, and Patient’s Global Assessment [PGA] of Disease Activity) that has historically been used in PsA clinical trials; however, DAPSA is a more valid measure of disease activity in patients with PsA as it includes the assessment of more joints (i.e., TJC 68 joints and SJC 66 joints) [25]. DAPSA also includes CRP, PGA of disease activity, and PGA of pain. Patient functional capability was assessed using the HAQ-DI [26] and the SF-36 PCS [27]. Mental health was assessed using the SF-36 MCS [27]. For PASI, DAS28, DAPSA, and HAQ-DI, a decrease in score indicates improvement [23–26]. For SF-36 MCS and PCS, an increase in score indicates improvement [27].

### Statistical analyses

#### Change in HRQoL and productivity measures by treatment group (discrimination)

Prespecified analyses that included all randomized patients were changed from baseline through Week 52 by treatment group for EQ-5D-5L index, EQ-VAS, daily productivity VAS, and WLQ productivity loss score. Change from baseline in EQ-5D-5L index, EQ-5D-5L dimension scores, EQ-VAS, daily productivity VAS, WLQ productivity loss score, and WLQ domain scores by baseline methotrexate (MTX) use through Week 52 were post hoc analyses that included all randomized patients. Treatment group comparisons for EQ-5D-5L index, EQ-VAS, daily productivity VAS, and WLQ productivity loss score at Weeks 14 and 24 were prespecified; all other treatment group comparisons were unplanned. Unadjusted *p* values of least squares mean differences between treatment groups through Week 24 were based on analysis of covariance, controlling for baseline score and baseline MTX usage. No formal comparisons were performed for time points after Week 24, when patients in the placebo group crossed over to golimumab, and there was no control group.

EQ-5D-5L index and dimension scores and EQ-VAS scores were based on observed data. Scores for daily productivity VAS, WLQ domains and productivity loss, PASI, DAS28, DAPSA, HAQ-DI, and SF-36 PCS and MCS were based on imputed data using last observation carried forward for missing data.

#### Correlation of change in HRQoL and productivity measures with change in disease activity and patient functional capability measures in the IV golimumab group

In patients randomized to the IV golimumab treatment group, Pearson correlation coefficient tests with Fisher’s transformed 95% confidence intervals were performed post hoc to evaluate the relationship between improvement from baseline in HRQoL (EQ-5D-5L index and EQ-VAS) and productivity outcomes (daily productivity VAS and WLQ productivity loss score) with underlying improvements in disease activity (PASI, DAS28, and DAPSA), patient functional capability (HAQ-DI and SF-36 PCS), and mental health (SF-36 MCS) outcomes.

#### Impact of clinical outcomes on patient utility

A multivariate analysis using a mixed-effect repeated measures model based on observed data until Week 24 (the placebo-controlled period) in the pooled patient population including both treatment groups was also conducted to quantify the impact of multiple attributes (age, gender, geographic region, PsA disease duration, PASI score, enthesitis, dactylitis, TJC 68, SJC 66, CRP, and HAQ-DI; independent variables) on utility, as measured by the EQ-5D-5L index (dependent variable). The independent variables were identified based on core outcome measures recommended by Outcome Measures in Rheumatology Clinical Trials (OMERACT) [7] and guideline utility mapping by the International Society for Pharmacoeconomics and Outcome Research (ISPOR) [28] and were included in the multivariate analysis based on univariate analyses and evaluation of collinearity between variables. Univariate analysis was first performed using a mixed-effect repeated measures model based on observed data until Week 24 in the pooled patient population to assess for association of attributes with the EQ-5D-5L index score. Variables were assessed for multicollinearity by variance inflation factor (VIF). A VIF of <5 was deemed acceptable [29]. Based on univariate analyses (*p*<0.20) and evaluation of collinearity between variables, all of the previously listed attributes were included in the multivariate models. In the multivariate analysis, attributes were dropped based on statistical significance to yield the most parsimonious model. Akaike information criterion (AIC) was calculated for all models. AIC is a measure based on in-sample fit to estimate the likelihood of a model to predict or estimate the future values. A smaller AIC reflects a better fit.

## Results

### Patient disposition and disease characteristics

A total of 480 patients were randomized to IV golimumab (*n*=241) or placebo (*n*=239) (Table 1). Patient disposition through Week 52 has been previously reported in detail [15]. Baseline patient demographic and disease characteristics were generally well balanced between treatment groups (Table 1). Mean age was 46 years, and 52% of all patients were men. The EQ-5D-5L index score was 0.6 in both groups, EQ-VAS was 46.2 in the placebo group and 46.9 in the golimumab group, daily productivity VAS was 5.9 in the placebo group and 6.1 in the golimumab group, and the WLQ productivity loss score was 8.8 in the placebo group and 9.3 in the golimumab group.

**Table 1:** GO-VIBRANT baseline demographics and disease characteristics of randomized patients

	Placebo	IV golimumab 2 mg/kg	Total
Randomized patients, *n*	239	241	480
Age, years	46.7 (12.5)	45.7 (11.3)	46.2 (11.9)
Male, *n* (%)	121 (50.6)	128 (53.1)	249 (51.9)
Race, *n* (%)			
White	237 (99.2)	241 (100)	478 (99.6)
Weight, kg	82.8 (17.9)	84.4 (21.1)	83.6 (19.6)
BMI, kg/m^2^	28.9 (6.2)	28.9 (6.4)	28.9 (6.3)
Duration of PsA, years	5.3 (5.9)	6.2 (6.0)	5.8 (6.0)
Methotrexate use, *n* (%)	173 (72.4)	163 (67.6)	336 (70.0)
SF-36 PCS, 0–100^a^	34.0 (7.2)	33.1 (6.9)	33.6 (7.1)
SF-36 MCS, 0–100^a^	42.5 (10.2)	43.5 (11.4)	43.0 (10.8)
HAQ-DI, 0–3	1.3 (0.6)	1.3 (0.6)	1.3 (0.6)
CRP, mg/dL	2.0 (2.1)	1.9 (2.5)	2.0 (2.3)
Number of swollen joints, 0–66	14.1 (8.2)	14.0 (8.4)	14.0 (8.3)
Number of tender joints, 0–68	26.1 (14.4)	25.1 (13.8)	25.6 (14.1)
DAPSA^a^	54.9 (22.8)	54.2 (21.6)	54.6 (22.2)
DAS28 (CRP)^a^	5.5 (1.1)	5.4 (1.0)	5.5 (1.0)
PASI score, 0–72^b^	8.9 (9.0)	11.0 (9.9)	9.9 (9.5)
EQ-5D-5L Index, 0 to 1^a,c^	0.6 (0.1)	0.6 (0.1)	0.6 (0.1)
EQ-VAS, 0–100 mm^a^	46.2 (20.3)	46.9 (20.1)	46.6 (20.2)
Daily productivity VAS, 0–10 cm^a^	5.9 (2.7)	6.1 (2.6)	6.0 (2.6)
WLQ productivity loss score, 0–100 mm^d^	8.8 (4.7)	9.3 (5.2)	9.0 (5.0)

All values are mean (standard deviation) unless otherwise noted.

^a^Placebo *n*=236, IV golimumab 2 mg/kg *n*=237, total *n*=473

^b^Among patients with ≥3% BSA psoriasis skin involvement at baseline; placebo *n*=188, IV golimumab 2 mg/kg *n*=189, total *n*=377

^c^Patients with severe disease may have values slightly below 0, representing a state they believe is worse than death

^d^Among patients who were working full or part time at baseline, including volunteering; placebo *n*=108, IV golimumab 2 mg/kg *n*=111, total *n*=219

*BMI*, body mass index; *BSA*, body surface area; *CRP*, C-reactive protein; *DAPSA*, Disease Activity index for PSoriatic Arthritis; *DAS28*, Disease Activity Score including 28 joints; *EQ-5D-5L*, EuroQol-5 dimension-5 level; *EQ-VAS, *EQ-5D-5L visual analog scale; *HAQ-DI*, Health Assessment Questionnaire-Disability Index; *IV*, intravenous; *MCS*, mental component summary; *n*, number of patients; *PASI*, Psoriasis Area and Severity Index; *PCS*, physical component summary; *PsA*, psoriatic arthritis; *SF-36*, Short Form 36 Health Survey; *VAS*, visual analog scale; *WLQ*, Work Limitations Questionnaire

### Change in HRQoL and productivity measures

#### EQ-5D-5L

**Fig 1:**
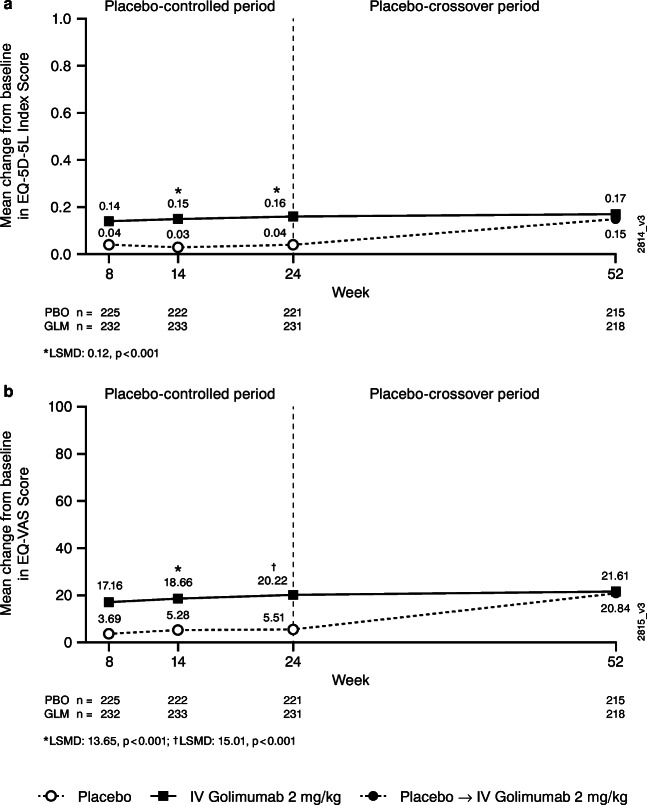
Mean change from baseline in EQ-5D-5L index (**a**) and EQ-VAS (**b**) scores through Week 52 in all randomized patients. Change from baseline is based on observed values. The adjusted *p* values are based on ANCOVA controlling for baseline MTX usage (yes, no) and baseline EQ-5D-5L index (**a**) or EQ-VAS (**b**) scores. *ANCOVA*, analysis of covariance; *EQ-5D-5L*, EuroQol-5 dimension-5 level; *EQ-VAS*, EQ-5D-5L visual analog scale; *GLM*, golimumab; *IV*, intravenous; *LSMD*, least square mean difference; *MTX*, methotrexate; *n*, number of patients; *PBO*, placebo

As early as Week 8 and through Week 24, patients randomized to IV golimumab had greater mean improvements in the EQ-5D-5L index than patients randomized to placebo (Week 8: 0.14 vs 0.04, respectively; Week 24: 0.16 vs 0.04, respectively) (Fig. 1a). At Week 52, after patients randomized to placebo had crossed over to IV golimumab for several months, the golimumab group and the placebo-crossover group had similar mean improvements from baseline (0.17 and 0.15, respectively). Results for the individual EQ-5D-5L dimension scores (i.e., mobility, self-care, usual activities, pain/discomfort, and anxiety/depression) were generally similar to those observed for the EQ-5D-5L index score, with greater mean improvements in each dimension score in the golimumab group versus the placebo group through Week 24 and similar improvement at Week 52 in the golimumab and placebo-crossover groups (Online Resource 1 Supplemental Fig. 1a–e).

#### EQ-VAS

Greater improvements in mean EQ-VAS scores in the IV golimumab group compared with the placebo group were also observed as early as Week 8 (17.16 vs 3.69, respectively) and maintained through Week 24 (20.22 vs 5.51, respectively; *p*<0.001) (Fig. 1b). At Week 52, improvement in EQ-VAS score from baseline was similar between the golimumab and the placebo-crossover groups (21.61 and 20.84, respectively).

#### Daily productivity VAS

**Fig 2:**
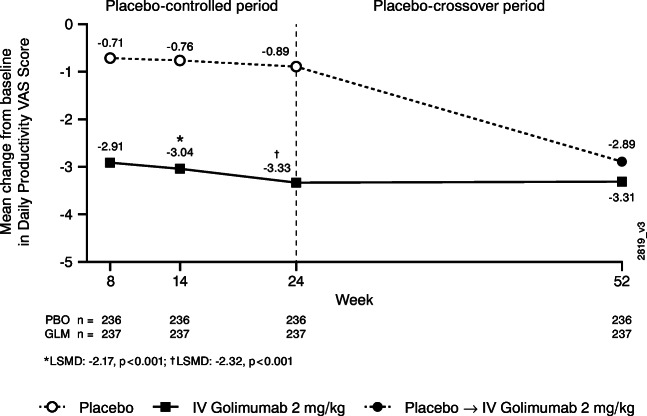
Mean change from baseline in daily productivity VAS score through Week 52 in all randomized patients. Change from baseline is based on imputed data using LOCF for missing data. Patients with no value at baseline are excluded from the analysis. The adjusted *p* values are based on ANCOVA controlling for baseline MTX use (yes, no) and baseline daily productivity VAS score. *ANCOVA*, analysis of covariance; *GLM*, golimumab; *IV*, intravenous; *LOCF*, last observation carried forward; *LSMD*, least square mean difference; *MTX*, methotrexate; *n*, number of patients; *PBO*, placebo; *VAS*, visual analog scale

Improvements in mean daily productivity VAS scores were greater in patients randomized to IV golimumab than in patients randomized to placebo as early as Week 8 (−2.91 vs −0.71, respectively) and maintained through Week 24 (−3.33 vs −0.89, respectively; *p*<0.001) (Fig. 2). At Week 52, improvements in mean daily productivity VAS scores were similar between the golimumab and placebo-crossover groups (−3.31 and −2.89, respectively).

#### Work Limitations Questionnaire

**Fig 3:**
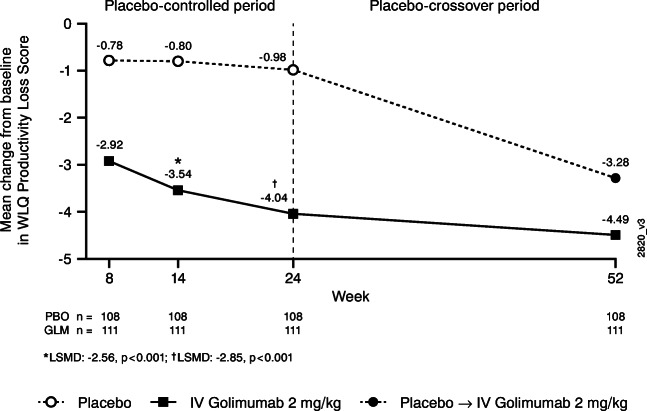
Mean change from baseline in WLQ productivity loss score through Week 52 among patients who were working or volunteering full or part time (*n*=219). Change from baseline is based on imputed data using LOCF for missing data. Patients with no value at baseline are excluded from the analysis. The adjusted *p* values are based on ANCOVA controlling for baseline MTX usage (yes, no) and baseline WLQ productivity loss score. *ANCOVA*, analysis of covariance; *LOCF*, last observation carried forward; *LSMD*, least square mean difference; *GLM*, golimumab; *IV*, intravenous; *MTX*, methotrexate; *n*, number of patients; *PBO*, placebo; *WLQ*, Work Limitations Questionnaire

Among the 219 (46%) patients (108 in the placebo group, 111 in the IV golimumab group) who were working or volunteering full or part time at baseline, improvements in mean WLQ productivity loss scores were greater in patients randomized to IV golimumab than in patients randomized to placebo as early as Week 8 (−2.92 vs −0.78, respectively) and maintained through Week 24 (−4.04 vs −0.98, respectively, *p*<0.001) (Fig. 3). At Week 52, improvements in mean WLQ productivity loss scores were similar between the golimumab and placebo-crossover groups (−4.49 and −3.28, respectively). Results for the individual WLQ domain scores (i.e., mental−interpersonal, output, physical demands, and time management) were comparable to those observed for the WLQ productivity loss score, with greater mean improvements in each domain score in the golimumab group versus the placebo group through Week 24 and similar improvement at Week 52 in the golimumab and placebo-crossover groups (Online Resource 1 Supplemental Fig. 2a–d).

#### Baseline methotrexate use

Overall, 70% of patients in this trial were using MTX at baseline (72% in the placebo group and 68% in the golimumab group). Results were similar for all assessments at each time point regardless of baseline MTX use, with a few exceptions (Online Resource 1 Supplemental Figs. 1–3). For the EQ-5D-5L anxiety/depression dimension score, in the golimumab group, the change from baseline through Week 52 was smaller in patients without versus with MTX use at baseline and, at Week 8, the change from baseline in the placebo group was smaller in patients with (−0.05) versus without (-0.17) baseline MTX use (Online Resource 1 Supplemental Fig. 1e). For EQ-VAS, in the placebo group, the change from baseline at Week 8 was smaller in patients with (2.22) versus without (7.23) MTX use at baseline (Online Resource 1 Supplemental Fig. 3b). For the WLQ mental–interpersonal domain score, in the placebo group, the change from baseline through Week 24 was smaller in patients with versus without baseline MTX use, and, in the golimumab group, improvement at Week 52 was smaller in patients without (−4.52) versus with (−13.52) baseline MTX use (Online Resource 1 Supplemental Fig. 2a). For this domain score, in patients without baseline MTX use, the change from baseline at Week 8 was greater in the placebo group compared with the golimumab group. Finally, for the WLQ output domain score, in the placebo group, improvement was smaller through Week 24 in patients with versus without baseline MTX use (Online Resource 1 Supplemental Fig. 2b).

### Correlation of change in HRQoL and productivity measures with disease activity and patient functional capability measures

Improvements from baseline in the EQ-5D-5L index, EQ-VAS, WLQ productivity loss, and daily productivity VAS scores were generally moderately correlated with improvements from baseline in DAPSA, DAS28, HAQ-DI, and SF-36 PCS and MCS (Table 2, Online Resource 1 Supplemental Table 1). Correlations were generally similar from Week 8 through Week 52; however, EQ-VAS had weaker correlations with disease activity and functional capability measures at Weeks 8 and 14. DAS28 generally had slightly stronger correlations with HRQoL and productivity measures compared with DAPSA. Improvements in PASI weakly correlated with improvements in all of the HRQoL and productivity measures.

**Table 2:** Correlation of improvement from baseline between HRQoL and productivity measures and disease activity and patient functional capability measures through Week 52 in patients randomized to receive IV golimumab

General health and productivity measures	Disease activity measures	Week 8			Week 14			Week 52		
		*n*	Pearson coefficient	95% CI	*n*	Pearson coefficient	95% CI	*n*	Pearson coefficient	95% CI
EQ-5D-5L index	DAPSA	232	-0.435	-0.533, -0.324	233	-0.399	-0.501, -0.284	218	-0.474	-0.570, -0.363
	DAS28	232	-0.412	-0.513, -0.298	233	-0.399	-0.501, -0.285	218	-0.513	-0.604, -0.407
	HAQ-DI	232	-0.608	-0.683, -0.519	233	-0.615	-0.688, -0.527	218	-0.641	-0.713, -0.555
	PASI	--	--	--	186	-0.066	-0.208, 0.079	173	-0.103	-0.248, 0.047
	SF-36 PCS	232	0.566	0.471, 0.647	233	0.538	0.439, 0.622	218	0.642	0.556, 0.713
	SF-36 MCS	232	0.510	0.407, 0.598	233	0.467	0.359, 0.561	218	0.460	0.347, 0.558
EQ-VAS	DAPSA	232	-0.278	-0.392, -0.154	233	-0.256	-0.372, -0.131	218	-0.387	-0.493, -0.267
	DAS28	232	-0.198	-0.318, -0.070	233	-0.244	-0.361, -0.118	218	-0.492	-0.586, -0.383
	HAQ-DI	232	-0.327	-0.437, -0.206	233	-0.289	-0.402, -0.166	218	-0.519	-0.609, -0.414
	PASI	--	--	--	186	-0.004	-0.148, 0.140	173	-0.009	-0.158, 0.140
	SF-36 PCS	232	0.384	0.268, 0.488	233	0.354	0.236, 0.461	218	0.608	0.515, 0.684
	SF-36 MCS	232	0.234	0.108, 0.352	233	0.132	0.003, 0.256	218	0.223	0.093, 0.345
Daily productivity VAS	DAPSA	237	0.365	0.249, 0.470	237	0.346	0.228, 0.453	237	0.325	0.205, 0.434
	DAS28	237	0.419	0.307, 0.518	237	0.436	0.326, 0.533	237	0.504	0.402, 0.593
	HAQ-DI	237	0.451	0.343, 0.547	237	0.534	0.435, 0.618	237	0.516	0.415, 0.603
	PASI	--	--	--	189	0.074	-0.070, 0.214	189	0.100	-0.044, 0.239
	SF-36 PCS	237	-0.407	-0.507, -0.294	237	-0.430	-0.528, -0.319	237	-0.517	-0.604, -0.416
	SF-36 MCS	237	-0.439	-0.536, -0.330	237	-0.436	-0.533, -0.326	237	-0.428	-0.526, -0.317
WLQ productivity loss	DAPSA	111	0.313	0.133, 0.471	111	0.277	0.094, 0.439	111	0.147	-0.041, 0.324
	DAS28	111	0.325	0.146, 0.481	111	0.349	0.173, 0.502	111	0.334	0.156, 0.489
	HAQ-DI	111	0.528	0.377, 0.649	111	0.601	0.465, 0.707	111	0.428	0.261, 0.568
	PASI	--	--	--	89	-0.005	-0.213, 0.204	89	0.083	-0.128, 0.286
	SF-36 PCS	111	-0.432	-0.571, -0.265	111	-0.428	-0.567, -0.260	111	-0.445	-0.581, -0.280
	SF-36 MCS	111	-0.515	-0.639, -0.362	111	-0.476	-0.607, -0.316	111	-0.478	-0.609, -0.318

WLQ daily productivity, WLQ productivity loss, SF-36 PCS and MCS, DAS28, HAQ-DI, and PASI scores were based on imputed data using LOCF for missing data. EQ-5D-5L index and EQ-VAS scores were based on observed data.

--, not available; *CI*, confidence interval; *DAPSA*, Disease Activity index for PSoriatic Arthritis; *DAS28*, Disease Activity Score including 28 joints; *EQ-5D-5L*, EuroQol-5 dimension-5 level; *EQ-VAS*, EQ-5D-5L visual analog scale; *HAQ-DI*, Health Assessment Questionnaire-Disability Index; *LOCF*, last observation carried forward; *MCS*, mental component summary; *n*, number of patients; *PASI*, Psoriasis Area and Severity Index; *PCS*, physical component summary; *SF-36*, Short Form 36 Health Survey; *VAS*, visual analog scale; *WLQ*, Work Limitations Questionnaire

### Impact of clinical outcomes on the EQ-5D-5L index

VIFs for all attributes originally included in this analysis (i.e., age, gender, geographic region, PsA disease duration, PASI score, enthesitis, dactylitis, TJC 68, SJC 66, CRP, and HAQ-DI) were ≤2.75; therefore, multicollinearity is not likely a concern (generally, a VIF >4 suggests multicollinearity). Since the univariate regressions showed that all attributes were associated with the EQ-5D-5L index score through Week 24 with a *p* value <0.20, they were all included in the multivariate analysis. In the final multivariate model, PASI score, enthesitis, TJC, CRP, and HAQ-DI were statistically significantly associated with the EQ-5D-5L index through Week 24 (Table 3). Based on the coefficient of each attribute in the final model, the impact of a 10-unit change in PASI score on the EQ-5D-5L index was similar to the impact of a 10-unit change in TJC or the presence of enthesitis.

**Table 3:** Association of clinical manifestations of psoriatic arthritis with the EQ-5D-5L index in a multivariate mixed-effect repeated measures model

Variables	Model 1		Model 2		Model 3		Model 4	
	β	*p* value	β	*p* value	β	*p* value	β	*p* value
Age, years	0.00030	0.3240	0.00031	0.2971	0.00031	0.2942		
Female	-0.00215	0.7640	--	--	--	--	--	--
Geographic region (EU vs NA)	-0.02449	0.1981	-0.02542	0.1780	-0.02595	0.1678	--	--
Duration of PsA, years	0.00016	0.7857	--	--	--	--	--	--
PASI score	-0.00123	0.0010	-0.00122	0.0010	-0.00121	0.0010	-0.00126	0.0006
Enthesitis (yes vs no)	-0.01199	0.0424	-0.01205	0.0412	-0.01205	0.0398	-0.01237	0.0348
Dactylitis (yes vs no)	-0.00083	0.8957	-0.00073	0.9088	--	--	--	--
TJC (0–68)	-0.00122	<0.0001	-0.00122	<0.0001	-0.00116	<0.0001	-0.00112	<0.0001
SJC (0–66)	0.00017	0.6990	0.00016	0.7070	--	--	--	--
CRP (mg/L)	-0.00078	<0.0001	-0.00078	<0.0001	-0.00077	<0.0001	-0.00079	<0.0001
HAQ-DI	-0.16670	<0.0001	-0.16690	<0.0001	-0.16670	<0.0001	-0.1664	<0.0001
Regression diagnostic								
AIC	-2661.8		-2665.6		-2669.5		-2670.3	

Based on 1367 observations.

AIC was similar for all models when attributes were omitted from the models.

--, not included in model; *AIC*, Akaike information criterion; *CRP*, C-reactive protein; *EQ-5D-5L*, EuroQol-5 dimension-5 level; *EU*, Europe; *HAQ-DI*, Health Assessment Questionnaire-Disability Index; *NA*, North America; *PASI*, Psoriasis Area and Severity Index; *PsA*, psoriatic arthritis; *SJC*, swollen joint count; *TJC*, tender joint count

## Discussion

The data presented here demonstrate that reducing disease activity has a positive impact on HRQoL; daily productivity at work, school, or home; and productivity at work in patients with PsA that is maintained through 1 year. As expected based on previous results in this patient population [14–16], in patients with active PsA, treatment with IV golimumab resulted in improvements in EQ-5D-5L index, EQ-VAS, daily productivity VAS, and WLQ productivity loss score as early as Week 8 that were maintained through Week 52. Although the type of work performed by the patients who were working at baseline was not recorded in the GO-VIBRANT trial, it should be noted that greater improvement compared with placebo was observed in all domains of the WLQ, including the physical demands domain, suggesting that improvement in productivity would be observed across a wide variety of occupations. Consistent results have also been observed in other randomized controlled trials of biologics that have evaluated work productivity outcomes, including daily productivity VAS, the Work Productivity Survey, and the WLQ index, as well as absenteeism and presenteeism, in patients with PsA [8, 11, 30, 31].

These results are also consistent with those from similar analyses of data from a randomized placebo-controlled trial of IV golimumab in patients with ankylosing spondylitis [17], suggesting that IV golimumab is effective in improving HRQoL and productivity in a variety of spondyloarthritides. In patients with ankylosing spondylitis, greater improvements with IV golimumab versus placebo in EQ-5D-5L index, EQ-VAS, daily productivity VAS, WLQ, and the ankylosing spondylitis quality of life questionnaire (ASQoL) were observed as early as Week 8 through Week 16, and improvements were maintained with golimumab treatment through Week 52 [17]. The data reported here and the data from patients with ankylosing spondylitis also provide further support for clinical trial discrimination of the measures tested. The daily productivity VAS is not frequently used in clinical practice or randomized controlled trials, but may be a very useful instrument in clinical practice.

Improvements in this trial were generally similar among patients who were and were not receiving concomitant MTX at baseline. However, it should be noted that this trial was not designed to detect differences between golimumab + MTX and golimumab alone and possible associations between longer-term golimumab persistence and concomitant MTX use are unknown.

In this study and in the similar study in ankylosing spondylitis discussed previously [17], weak-to-moderate correlations of measures of HRQoL and productivity with measures of disease activity and patient functional capability, as assessed by the HAQ-DI, were observed, demonstrating that these domains are related, but that improvement is not completely mediated by measured symptoms alone. However, there may be benefits of suppression of TNF and reduction in systemic inflammation that may be measured by these HRQoL and productivity instruments that may not be fully reflected in clinical measures of disease activity and patient functionality alone.

Although correlations between improvement in PASI score and improvement in HRQoL and productivity measures were weak, our multivariate analysis showed that absolute PASI score was significantly associated with absolute EQ-5D-5L index score through Week 24. The multivariate analysis assessed association between absolute values and, therefore, is not bound by treatment effect, making it an inherently clearer measure of association between disease activity and EQ-5D-5L index. In addition, correlation is not adjusted for scale and collinearity is not taken into account; thus, it is used here to ‘eyeball’ relationships between improvement in HRQoL and productivity measures and improvement in measures of disease activity and patient functionality, which in this particular trial, are bound by patient responses to IV golimumab.

A limitation of these data is that the results were observed in the context of a randomized controlled trial in patients with very high disease activity who predominantly have polyarticular disease. This may limit the generalizability of the observations to patients with less severe disease. Additionally, the type of work or hours of work per week may influence whether or not someone enrolls in a clinical trial; thus, there may be selection bias that should be considered when evaluating work outcomes. It should also be noted that the value set used for the EQ-5D-5L index score calculation in this study was based on the published United States value set for EQ-5D-5L [32]. Comparative exploration of differing EQ-5D-5L value sets by country may be explored in further analyses, but is beyond the scope of this current study. Further, the fact that the WLQ was only completed by patients who were working or volunteering at baseline does not allow for the evaluation of patients who may have started working following treatment during the study and should be considered when interpreting the WLQ results. The lack of information regarding whether patients were not working at baseline due to their disease and the type of work being done by patients who were working at baseline are also limitations of the trial.

In conclusion, PsA has a negative impact on HRQoL and productivity, and improvement in overall HRQoL and restoration of ability to engage productively in work and other activities remain an important goal of PsA treatment. In this trial, treatment with IV golimumab resulted in wide-ranging improvements in HRQoL and productivity, and these improvements appear to be associated with improvements in traditional measures of disease activity and patient functional capability.

## Supplementary Information

ESM 1 (DOCX 591 kb)

## Data Availability

The data sharing policy of Janssen Pharmaceutical Companies of Johnson & Johnson is available at https://www.janssen.com/clinical-trials/transparency. As noted on this site, requests for access to the trial data can be submitted through Yale Open Data Access (YODA) Project site at http://yoda.yale.edu.
