# Supplementary material for: The effect of intravenous golimumab on health-related quality of life and work productivity in patients with active psoriatic arthritis: results of the Phase 3 GO-VIBRANT trial
Source: Clin Rheumatol. 2021 Mar 2;40(9):3667–77. doi: 10.1007/s10067-021-05639-1 (PMC8357705; doi:10.1007/s10067-021-05639-1)
Supplement: Supplementary file 1 — (DOCX 591 kb) [file 10067_2021_5639_MOESM1_ESM.docx]

**The effect of intravenous golimumab on health-related quality of life and work productivity in patients with active psoriatic arthritis: results of the Phase 3 GO-VIBRANT trial**

**Authors:** Alexis Ogdie, MD, MSCE^1^, Jessica A. Walsh, MD^2^, Soumya D. Chakravarty, MD, PhD^3,4^, Steven Peterson, MPPM, MPH^5^, Kim Hung Lo, PhD^6^, Lilianne Kim, PhD^6^, Nan Li, PhD^7^, Elizabeth C. Hsia, MD, MSCE^1,6^, Eric K. H. Chan, PhD^7^, Arthur Kavanaugh, MD^8^, M. Elaine Husni, MD, MPH^9^

**Corresponding Author**

Alexis Ogdie, MD, MSCE

University of Pennsylvania

alogdie@pennmedicine.upenn.edu

**Supplemental Fig 1** Mean change from baseline in EQ-5D-5L mobility (a), self-care (b), usual activities (c), pain/discomfort (d), and anxiety/depression (e) dimension scores through Week 52 in all randomized patients and patients with/without baseline MTX use

Change from baseline is based on observed values.

The adjusted p-values are based on ANCOVA controlling for baseline MTX usage (Yes, No) and baseline scores.

ANCOVA, analysis of covariance, EQ-5D-5L, EuroQol-5 dimension-5 level; GLM, golimumab; IV, intravenous; LSMD, least square mean difference; MTX, methotrexate; n, number of patients; PBO, placebo

**Supplemental Fig 2** Mean change from baseline in WLQ mental-interpersonal (a), output (b), physical demands (c), and time management (d) domain scores through Week 52 in all randomized patients and in patients with and without baseline MTX use

Change from baseline is based on imputed data using LOCF for missing data; patients with no value at baseline were excluded from the analysis.

The adjusted p-values are based on ANCOVA controlling for baseline MTX usage (Yes, No) and baseline scores.

ANCOVA, analysis of covariance; GLM, golimumab; IV, intravenous; LOCF, last observation carried forward; LSMD, least square mean difference; MTX, methotrexate; n, number of patients; PBO, placebo; WLQ, Work Limitations Questionnaire

**Supplemental Fig 3** Mean change from baseline in EQ-5D-5L index (a), EQ-VAS (b), daily productivity VAS (c), WLQ productivity loss (d) scores through Week 52 in all randomized patients and in patients with/without baseline MTX use

For EQ-5D-5L index and EQ-VAS scores, change from baseline is based on observed values. For daily productivity VAS and WLQ productivity loss scores, change from baseline is based on imputed data using LOCF for missing data; patients with no value at baseline were excluded from the analysis.

The adjusted p-values are based on ANCOVA controlling for baseline MTX usage (Yes, No) and baseline scores.

ANCOVA, analysis of covariance; EQ-5D-5L, EuroQol-5 dimension-5 level; EQ-VAS, EQ-5D-5L visual analog scale; GLM, golimumab; IV, intravenous; LOCF, last observation carried forward; LSMD, least square mean difference; MTX, methotrexate; n, number of patients; PBO, placebo; VAS, visual analog scale; WLQ, Work Limitations Questionnaire

**Supplemental Table 1**. Correlation of improvement from baseline between HRQoL and productivity measures and disease activity and patient functional capability measures at Week 24 in patients randomized to receive IV golimumab

| **General Health and Productivity Measures** | **Disease Activity Measures** | **Week 24** | | |
| --- | --- | --- | --- | --- |
|  |  | **n** | **Pearson Coefficient** | **95% CI** |
| EQ-5D-5L Index | DAPSA | 231 | -0.330 | -0.439, -0.209 |
|  | DAS28 | 231 | -0.402 | -0.504, -0.287 |
|  | HAQ-DI | 231 | -0.679 | -0.742, -0.602 |
|  | PASI | 184 | -0.039 | -0.182, 0.107 |
|  | SF-36 PCS | 231 | 0.570 | 0.474, 0.650 |
|  | SF-36 MCS | 231 | 0.492 | 0.387, 0.583 |
| EQ-VAS | DAPSA | 231 | -0.287 | -0.401, -0.164 |
|  | DAS28 | 231 | -0.351 | -0.459, -0.232 |
|  | HAQ-DI | 231 | -0.411 | -0.512, -0.296 |
|  | PASI | 184 | 0.012 | -0.133, 0.156 |
|  | SF-36 PCS | 231 | 0.416 | 0.302, 0.516 |
|  | SF-36 MCS | 231 | 0.258 | 0.132, 0.374 |
| Daily Productivity VAS | DAPSA | 237 | 0.306 | 0.185, 0.417 |
|  | DAS28 | 237 | 0.423 | 0.311, 0.521 |
|  | HAQ-DI | 237 | 0.581 | 0.489, 0.659 |
|  | PASI | 189 | 0.087 | -0.056, 0.227 |
|  | SF-36 PCS | 237 | -0.503 | -0.592, -0.401 |
|  | SF-36 MCS | 237 | -0.405 | -0.506, -0.292 |
| WLQ Productivity Loss | DAPSA | 111 | 0.182 | -0.005, 0.356 |
|  | DAS28 | 111 | 0.241 | 0.056, 0.408 |
|  | HAQ-DI | 111 | 0.506 | 0.351, 0.632 |
|  | PASI | 89 | 0.050 | -0.161, 0.255 |
|  | SF-36 PCS | 111 | -0.487 | -0.616, -0.329 |
|  | SF-36 MCS | 111 | -0.360 | -0.511, -0.185 |

WLQ daily productivity, WLQ productivity loss, SF-36 PCS and MCS, DAS28, HAQ-DI, and PASI scores were based on imputed data using LOCF for missing data. EQ-5D-5L index and EQ-VAS scores were based on observed data.

CI, confidence interval; DAPSA, Disease Activity index for PSoriatic Arthritis; DAS28, Disease Activity Score including 28 joints; EQ-5D-5L, EuroQol-5 dimension-5 level; EQ-VAS, EQ-5D-5L visual analog scale; HAQ-DI, Health Assessment Questionnaire-Disability Index; HRQoL, health-related quality of life; LOCF, last observation carried forward; MCS, mental component summary; n, number of patients; PASI, Psoriasis Area and Severity Index; PCS, physical component summary; SF-36, Short Form 36 Health Survey; VAS, visual analog scale; WLQ, Work Limitations Questionnaire
